# Comparative proteome and peptidome analysis of the cephalic fluid secreted by *Arapaima gigas* (Teleostei: Osteoglossidae) during and outside parental care

**DOI:** 10.1371/journal.pone.0186692

**Published:** 2017-10-24

**Authors:** Lucas S. Torati, Hervé Migaud, Mary K. Doherty, Justyna Siwy, Willian Mullen, Pedro E. C. Mesquita, Amaya Albalat

**Affiliations:** 1 EMBRAPA Fisheries and Aquaculture, Palmas-Tocantins, Brazil; 2 Institute of Aquaculture, University of Stirling, Stirling, Scotland, United Kingdom; 3 Proteome Analysis Facility, University of the Highlands and Islands, Inverness, United Kingdom; 4 Mosaiques Diagnostics GmbH, Hannover, Germany; 5 College of Medical, Veterinary and Life Sciences, University of Glasgow, Glasgow, Scotland, United Kingdom; 6 Center of Research in Aquaculture Rodolpho von Ihering, CPA/DNOCS, Ombreira Direita, Pentecoste, Ceará, Brazil; Pacific Northwest National Laboratory, UNITED STATES

## Abstract

Parental investment in *Arapaima gigas* includes nest building and guarding, followed by a care provision when a cephalic fluid is released from the parents’ head to the offspring. This fluid has presumably important functions for the offspring but so far its composition has not been characterised. In this study the proteome and peptidome of the cephalic secretion was studied in parental and non-parental fish using capillary electrophoresis coupled to mass spectrometry (CE-MS) and GeLC-MS/MS analyses. Multiple comparisons revealed 28 peptides were significantly different between males and parental males (PC-males), 126 between females and parental females (PC-females), 51 between males and females and 9 between PC-males and PC-females. Identification revealed peptides were produced in the inner ear (pcdh15b), eyes (tetraspanin and ppp2r3a), central nervous system (otud4, ribeye a, tjp1b and syn1) among others. A total of 422 proteins were also identified and gene ontology analysis revealed 28 secreted extracellular proteins. From these, 2 hormones (prolactin and stanniocalcin) and 12 proteins associated to immunological processes (serotransferrin, α-1-antitrypsin homolog, apolipoprotein A-I, and others) were identified. This study provides novel biochemical data on the lateral line fluid which will enable future hypotheses-driven experiments to better understand the physiological roles of the lateral line in chemical communication.

## Introduction

Freshwater oviparous teleosts lay eggs in rivers and lakes where food resources are not always abundant and predators or pathogens can significantly impact numbers of descendants. Along the evolution of teleosts, several mechanisms have arisen comprising parental investment, which include parental actions to increase offspring survival [1]. Parental care behaviour in teleosts involves building nests with appropriate substrates, defending and guarding the eggs, embryos and larvae against predators, and in some species parents provide protection for extended periods during juvenile development [2]. An important component of parental care strategies is the provision of nutrients to the offspring either through egg yolk or more rarely at post-hatch stages (e.g. mucus feeding) [3]. In some species, passive immunity or stimulated growth can occur via parental biochemical interaction with the offspring [4]. The provision of nutrients, growth and immunity factors at early developmental stages is particularly important to reduce the time offspring spend under more vulnerable conditions, therefore increasing likelihood of survival.

*Arapaima gigas* (Schinz, 1822) is a large fish characterised by late sexual maturity, small clutches and high parental investment per offspring [5]. Despite being an emblematic species of the Neotropical ichthyofauna (adults can reach up to 3 m in length) [6], the species is threatened due to overexploitation of natural stocks combined with the lack of knowledge on its basic biology [7]. Reproduction of *A*. *gigas* in natural environments begins with the rainy season generally from December to May [8]. With the start of the flooding, adults build nests for mating in temporary lagoons [8]. Prior to reproduction, chasings and fights indicate marked territorialism in the species, and spawning is followed by external fertilization on the nest [9]. After spawning, the male and female initiate nest guarding behaviour: while one parent is at the surface breathing, the other is always protecting the brood on the nest. The occurrence of mouth incubation or egg transportation has been suggested [10], but no systematic observation supporting oral incubation in *A*. *gigas* has been reported. Nine days after spawning, the eggs hatch and larvae start air-breathing. The male provides an intensive parental care which can last up to 3 months, guiding the offspring above its darkened head into zooplankton-rich areas for feeding [11]. Fry shoaling at this stage is remarkably organized, for instance the male darkening is believed to provide camouflage for the offspring against predators [12]. Female participation in parental care at this stage seems less relevant due to female unchanged body colour [13]. However, the female swims round the male and offspring at longer distances (>2m) for some period after the nest guarding phase [10]. This behaviour is still poorly understood, but could involve territorial inspection aiming at predator avoidance or location of food-rich areas [13]. The female normally leaves the male and offspring after a period not well documented (*cc*. 1 month), and can reproduce again with other males during the same reproductive season [8].

The mechanisms by which parental investment occur in *A*. *gigas* are not fully understood, and a particularity has called attention of many ichthyologists. On the head surface of adult males and females, the cephalic canals of the lateral line system are well developed forming a series of cavities covered by a pore-bearing integument, from which a whitish fluid is released into the water [14]. The indigenous refer to this fluid as “arapaima milk” since after fertilization on the nest, eggs, developing larvae and the growing offspring are in constant interaction with the male and female’s head, being in close contact with this secretion. Several roles for this fluid have been suggested since its production is intensified during reproduction and parental care. However, whether the cephalic secretion contributes to fingerling nutrition [15–18] or enhances the fry immunological system increasing survival rates [19] are hypotheses never truly investigated given the lack of basic information on the biochemical nature of the cephalic secretion. Lack of biochemical information hinders also our understanding on fish chemical communication, which involves release of pheromones and signature mixtures, that convey varied messages into conspecifics including the offspring [20].

In this context, the un-targeted study of the proteome and peptidome is an ideal “search space” for molecules which can help us to understand the functions behind biological fluids, with examples found in proteomic studies of cerebrospinal fluids (CSF), fish mucus, plasma, tear fluid, urine and others [21–23]. With the analytical—omics advances of recent years, proteomes and peptidomes have been studied in different species, generating protein databases that are available for protein identification, phylogenetic comparisons and gene ontology analyses based on homology-driven approaches [24]. Consequently, proteomic methods are being used in a wide range of disciplines such as behavioural ecology, aquaculture and food sciences [25, 26]. In most cases, the initial mapping of proteins and peptides is necessary to allow later mechanistic driven hypotheses. Such examples are illustrated in studies with other parental fish species, such as the mapping of the mucus proteome in the discus fish (*Symphysodon aequifasciata*) [27], which later allowed better understanding on the roles of prolactin (PRL) in the parental care of that species [28]. Similarly, after an initial mapping of the mucus proteome of the Atlantic cod (*Gadus morhua*), important proteins related to fish immune response were elucidated [29].

In this study, peptides and proteins present in the cephalic fluid of arapaima comprising the anterior lateral line were analysed using different analytical platforms. Peptides were profiled using capillary electrophoresis coupled to mass spectrometry (CE-MS) and identifications were performed using liquid chromatography coupled to tandem mass spectrometry (LC-MS/MS) [30]. On the other hand, proteins present in the cephalic fluid were analysed using one-dimensional sodium dodecyl sulfate-polyacrylamide gel electrophoresis followed by liquid chromatography-tandem mass spectrometry (GeLC-Ms/MS) [31]. The combination of CE-MS and LC-MS/MS for the analysis of peptides in complex samples has been shown to be a reproducible and very sensitive platform [30] while GeLC-MS/MS is a robust technique commonly used to identify proteins in complex samples [31]. Having access to few of these valuable cephalic fluid samples during parental care of *A*. *gigas* enabled this study to characterise the proteome and peptidome comparing parental and non-parental males and females, thus generating novel data to increase our understanding of parental care processes in this species and possible roles of the cephalic secretion in teleosts.

## Materials and methods

### Animal sampling

This study was carried out in the *Rodolpho von Ihering* research station- DNOCS (3°48’09.54”S, 39°15’56.73”W) at Pentecoste (Brazil). Adult broodstock of *A*. *gigas* known to be over 6 years of age received a passive integrated transponder (PIT; AnimallTAG, São Carlos, Brazil) in the dorsal muscle to allow individual identification. Fish gender was identified with a vitellogenin enzyme immune assay (EIA) kit (Acobiom, Montpellier, France) developed specifically for *A*. *gigas* [32] and females were paired with males in 300m^2^ breeding earthponds (1–2 m depth). Along the study, fish were fed once a day *ad libitum* with 160 g floating balls made with a commercial ration (38% crude protein, Aquamix, Brazil) mixed with 10% tilapia flesh (*Oreochromis niloticus*). Welfare of this studied broodstock and rearing system have been previously described [33].The sampled males measured 162.8 ± 25.9 cm in total length (TL) and weighed 39.9 ± 16.8 kg, whilst females measured 180.5 ± 14.5 cm in TL and weighed 52.3 ± 12.3 kg. Before sampling, fish were fasted for 24 hours, netted from earthponds and kept contained on a soft wet mat for between 5–10 minutes. Anaesthetics were not applied during sampling as anaesthesia has been shown to compromise welfare and result in mortalities in *A*. *gigas* due to its obligate air-breathing behaviour [34]. Fish breathing behaviour was closely monitored during sampling (breathing at regular intervals of 4–6 minutes). Tags were read and 1–2 ml of cephalic fluid was sampled from the dorsalmost lateralis sensorial cavity of the preopercle using a sterile needle inserted inside the integument pocket (BD Precisionglide 22G, New Jersey, USA). The collected fluid did not include visible blood contamination. Samples were immediately frozen in liquid nitrogen and then stored at -80°C. After sampling, fish were returned to the ponds and monitored until return to normal breathing behaviour, and no mortality was recorded following this sampling procedure.

Non-parental and parental fish were sampled at different time points. A total of 11 non-parental males (males) and 12 non-parental females (females) were sampled in March 24^th^ 2014. Additionally, two couples spawned twice from March to June 2014, and from them a total of 10 samples were taken along the parental care phase. Since it was not possible to observe spawning of *A*. *gigas* due to water turbidity in breeding ponds, the fertilization time in *A*. *gigas* is calculated by subtracting 9 days from the day when offspring are first observed breathing [13]. Therefore, samples from parental males (PC-males) and females (PC-females) correspond to approximately 13, 25, 35 and 62 days post-spawning (dps). All samples were individually analysed in the capillary electrophoresis (CE-MS) after peptide extraction. For the protein analysis (GeLC-MS/MS), equal protein amounts of each sample were pooled after protein quantification. These protein pools corresponded to: males (n = 6 males at 1-time point), females (n = 6 females at 1-time point), PC-males (n = 5 samples, 1 male at 2-time points and 1 male at 3-time points) and PC-females (n = 5 samples, 1 female at 2-time points and 1 female at 3-time points).

This research complied with the “Brazilian guidelines for the care and use of animals for scientific and educational purposes”–DBCA, and this research was approved by the Ethics Committee for the Use of Animals—CEUA of the National Research Center on Fisheries, Aquaculture and Agricultural Systems—CNPASA (specific protocol n°09). Samples were shipped in dry ice for protein and peptide extraction at the University of Stirling (Stirling, Scotland) (Permit IBAMA/CITES n° 14BR015850/DF). Extracted peptides were analysed at the University of Glasgow and proteins at the University of the Highlands and Islands.

### Analysis of peptides

#### Extraction

All buffers and solutions used were freshly prepared, following the method from Albalat, Franke [35]. Cephalic fluid samples were thawed on ice and 800 μl used for peptide extraction. An initial centrifugation at 2,000 g (5 min; 4°C) was used to settle debris and impurities down. The supernatant was removed and centrifuged at 16,000 g (10 min 4°C) for delipidation. Following, 750 μL of the supernatant was removed and mixed with 750 μL of urea buffer (2 M urea, 100 mM NaCl, 10 mM NH_4_OH containing 0.01% SDS). This mixture was filtered in a 20 kD Centrisart tube (Sartorius Stedim, Geottingen, Germany), and centrifuged at 2,000 g to obtain 1.1 mL of filtrate. This filtrate was applied into a NAP -5 Saphadex desalting column (GE Healthcare Life Sciences, Buckinghamshire, UK) previously equilibrated with 25 ml of NH_4_ buffer (0.01% NH_4_OH in HPLC-grade H_2_O, pH 10.5–11.5), and eluted with 2 ml of NH_4_ buffer, collected in clean 5 mL polypropylene tubes, lyophilized and kept at 4°C until CE-MS analysis was performed.

#### Capillary electrophoresis (CE-MS)

Peptides were analysed with a P/ACE MDQ capillary electrophoresis system (Beckman Coulter, Brea, CA, USA) equipped with a capillary PicoTip Emitter TaperTip of 90 cm, 360 μm OD and 50 μm ID (New Objective, Woburn, USA) online coupled to a MS micro-TOF (Bruker Daltonics, Leipzig, Germany). Prior to analysis, the capillary was conditioned for 10 minutes at 50 psi with 1 M NaOH, and later for 20 minutes with running buffer (20% acetonitrile (ACN), 0.94% formic acid and 79.05% HPLC graded H_2_O). The MS was calibrated with a tuning solution containing lysozyme (14,303 Da), ribonuclease (13,681 Da), aprotinin (6,513 Da) and the following synthetic peptides: ELMTGELPYSHINNRDQIIFMVGR (2,832 Da), TGSLPYSHIGSRDQIIFMVGR (2,333 Da), GIVLYELMTGELPYSHIN (2,048 Da) and REVQSKIGYGRQIIS (1,733 Da) and analysis was made in reverse mode. Lyophilised peptide samples were reconstituted in 9 μl of HPLC grade water (Roth, Karlsruhe, Germany) and centrifuged at 12,000 g for 10 min at 4°C. Samples were injected at a pressure of 2 psi for 99s loading a volume of 290 nL. The capillary temperature was set at 25°C with separation made at 25 kV for 30 minutes. Sheath flow liquid (30% 2-propanol, 0.4% formic acid and 69.6% deionized water) was applied at the capillary end with a running speed of 0.02 ml/h. The ESI sprayer (Agilent Technologies, Palo Alto, CA, USA) was grounded (0 V), so the ion spray interface potential was set in -4.5 kV. The MS spectra were accumulated every three seconds, over a range of m/z of 50 to 3000 along 60 minutes. This method has been comprehensively described in [36] and [37]. Repeatability and reproducibility of CE-MS for peptide analysis has been reported in Mischak, Vlahou [38]. Validation of identified peptides was achieved by correlation between peptide change at the working pH of 2 and CE-migration time as shown in Zürbig, Renfrow [39].

#### LC-MS/MS analysis

The extracted peptides were also analysed with a Dionex Ultimate 3000 RSLS nano-flow system (Dionex, Camberly UK). The samples (5 μL) were loaded onto a Dionex 100 μm × 2 cm 5 μm C18 nano-trap column at a flowrate of 5 μL/min by an Ultimate 3000 RS autosampler (Dionex, Camberley UK). The composition of the loading solution was 0.1% formic acid and ACN (98:2). Once loaded onto the trap column the sample was washed off into an Acclaim PepMap C18 nano-column 75 μm × 15 cm, 2 μm 100 Å at a flowrate of 0.3 μL/min. The trap and nano-flow column were kept at 35°C in a column oven in the Ultimate 3000 RSLC. The samples were eluted with a gradient of solvent A: 0.1% formic acid and ACN (98:2) versus solvent B: 0.1% formic acid and ACN (20:80) starting at 5% B rising to 50% B over 100 min. The column was washed using 90% B before being equilibrated prior to the next sample being loaded.

The eluant from the column was directed to a Proxeon nano-spray ESI source (Thermo Fisher Hemel UK) operating in positive ion mode then into an Orbitrap Velos FTMS. The ionisation voltage was 2.5 kV and the capillary temperature was 200°C. The mass spectrometer was operated in MS—MS mode scanning from 380 to 2000 amu. The top 20 multiply charged ions were selected from each full scan for MS—MS analysis, the fragmentation method was HCD at 35% collision energy. The ions were selected for MS2 using a data dependent method with a repeat count of 1 and repeat and exclusion time of 15 s. Precursor ions with a charge state of 1 were rejected. The resolution of ions in MS 1 was 60,000 and 7500 for HCDMS2.

#### Data processing, peptide identification and statistical analysis

CE-MS raw data were processed for peak peaking, deconvolution and deisotoping using MosaiquesVisu software v. 2.1.0 (Mosaiques Diagnostics GmbH, Hannover, Germany). The threshold of signal to noise (SNR) was set at 4, and only signals present in 3 consecutive spectra were accepted. A matched filtering algorithm assigned charge based on the isotopic distribution. It generated a list containing all the interpretable signals with their *m/z*, migration time, charge, and signal intensity (ion counts). Signals representing the same compound with different charge states were then combined, generating a final list with compound identities defined by mass, migration time and relative abundance (ion count). To allow comparisons among different samples, CE migration time and ion signal intensity were normalized, resulting in a list with molecular mass (Da) and normalized CE migration time (min) for each feature. The normalized signal intensity was used to measure and compare the relative abundance among samples.

LC-MS/MS data were processed initially uploading the raw spectra data into Thermo Proteome Discoverer 1.2 Thermo Scientific (Hemel Hempstead, UK). Peak picking was performed under default settings for FTMS analysis such that only peptides with signal to noise ratio higher than 1.5 and belonging to precursor peptides between 700–8,000 Da were considered. Peptide and protein identification was performed with SEQUEST algorithm. An in house compiled database containing proteins from the latest version of the UniProt SwissProt database was compiled to include only *Danio rerio* entries. No enzyme cleavage was selected and oxidation of methionine and proline was chosen as variable modifications. Precursor tolerance was set at 20 ppm and 0.1 Da for MS/MS fragment ions. Resulting peptides and protein hits were further screened by excluding peptides with an error tolerance higher than 10 ppm and by accepting only those hits listed as high confidence by Proteome Discoverer software. Theoretical migration times in CE—MS for any resulting peptides were calculated so that sequences obtained with LC-MS/MS could be subsequently assigned to a position in the CE—MS analysis.

Aiming to discriminate peptide peaks and compare different groups, a non-parametric Wilcoxon signed rank test was conducted in R version 2.15.3 [40], and corrected for false-discovery rate with Benjamini and Hochberg (BH) procedure [41]. Based on Wilcoxon test, significantly different (p<0.05) peptides among groups were identified using LC-MS/MS dataset (S1 Table) following Zürbig, Renfrow [39]. In this process, peptides data obtained with the CE-MS had their theoretical migration times, MW and number of basic amino acids (aa) matched with LC-MS/MS peptide list, enabling identification using a threshold of 80 ppm on MW. This procedure has been successfully used in several studies linking specific CE-MS-identified peptides to sequences obtained with LC-MS/MS [39].

Using the complete catalogue of peptides detected through the LC-MS/MS, Gene Ontology (GO) analyses were performed using STRAP *v 1*.*5*. software [42]. Also, aiming to identify secreted (extracellular) peptides, a GO analysis was also made for those peptides significantly different among groups after unadjusted Wilcoxon testing.

### Analysis of proteins by GeLC-MS/MS

#### Extraction and quantification

Initially, the remaining cephalic secretion was centrifuged at 2,000 g for 5 minutes at 4°C to remove any debris. Given low protein levels in samples, a protein precipitation was made at 1:3 v/v secretion/acetone (HPLC grade, Fisher Scientific). The mixture was cooled down to -20°C for 2 hours, then centrifuged at 5,000 g for 40 minutes at 4°C. The supernatant was discarded, the protein pellet dried under a N_2_ stream and reconstituted in non-reducing buffer (13.1 mM Tris—pH 6.8, 2.63% v/v Glycerol, 0.42% v/v SDS). An aliquot was taken for protein quantification whilst the extracts were kept at -80°C until inGeLC-MS/MS analysis.

The total protein content was quantified using a bicinchoninic acid assay kit (BCA) (Uptima UP40840B, Interchim, France). Reactions were prepared in 96 well plates using 2 μl of extracted sample in 40 μl of working solution (1:20 reaction) following the kit protocol. The plate was incubated for 30 minutes at 37°C, cooled at room temperature and read at 562 nm using a ND-1000 NanoDrop Spectrophotometer (Thermo Fisher Scientific, DE, USA). A serial dilution of bovine serum albumin (10–2,000 μg/ml) was used to prepare a standard curve against which the sample readings were interpolated.

#### In-gel digestion

Aiming to substantially expand protein identifications following Paulo [43], each pool of samples (males, females, PC-males and PC-females) was analysed with three technical replicates. Samples (10 μg of total protein per pool) were mixed in reducing buffer (13.1 mM Tris—pH 6.8, 2.63% v/v Glycerol, 0.42% v/v SDS, 0.243% v/v bromophenol blue and 163.5 mM DTT), heated up to 95°C for 5 minutes on a heating block and centrifuged at 2,000 g for 30 seconds. Reduced lysates were loaded into a 1D SDS polyacrylamide gel (4–20%, Mini-PROTEAN TGX, BIO-RAD) with a protein ladder reference (5 μl, BenchMark, 10-220kDa, ThermoFisher Scientific). Gels were run in a BIO-RAD Mini-PROTEAN Vertical Electrophoresis apparatus immersed in tris-glycine running buffer (24.7 mM Tris-Base, 191.8 mM glycine, 3.46 mM SDS, ultra-pure water, pH adjusted to 8.3) at 200V (400 mA) for 25 minutes until the frontline reached 2.5 cm (incomplete run). A compete run of these pools was made with the purpose of band visualization (graphical abstract). The run gels were fixed in 200 ml of fixation solution (7% v/v acetic acid, 40% v/v methanol) for 1 hour at room temperature. Following, fixation solution was discarded and gel washed 3 times (5 minutes each) with distilled water (200 ml). Gels were stained with 25 ml of Comassie G-250 (SimplyBlue Safe Stain, Thermo Fisher Scientific) for 45 minutes at room temperature. After discarding the stain, gels were washed and rinsed in ultra-pure water for 1 hour. Under a laminar flow cabinet, each gel lane was sliced in 10–12 cuts (plugs) of approximately 2.0–2.5 mm each, then processed independently in 1.5 ml reaction polypropylene tubes. For destaining, plugs were incubated twice (10 minutes each) in 100 μl of destaining solution (50 mM ammonium bicarbonate and ACN 50% v/v) at 37°C. Samples were then incubated in 50 μl of reducing solution (10 mM dithiothreitol-DTT and 100 mM ammonium bicarbonate) at 37°C for 30 minutes. Following, plugs were incubated in the dark with 50μl of alkylation solution (55.1 mM iodoacetamide, 100 mM ammonium bicarbonate) at 37°C for 30 minutes, and then dehydrated in 100 μl of ACN at 37°C for 15 minutes. After complete ACN evaporation on a heat block (37°C), plugs were digested with 50 μl of trypsin (Promega, Madison, USA) prepared in a solution of 5 mM acetic acid and 45 mM ammonium bicarbonate. Initially, samples were incubated at 37°C for 1 hour. Then, 20 μl of 100 mM ammonium bicarbonate was added and reaction allowed for 14 hours at 37°C. Finally, the digests were transferred into sterile 1.5 mL polypropylene tubes containing 2 μl of 10% formic acid, and frozen at -20°C until analysis.

#### LC-MS/MS analysis

Tryptic digests were analysed with a LTQ-Orbitrap XL LC−MS^n^ mass spectrometer (Thermo Fisher Scientific, Bremen, Germany) equipped with a nanospray source and coupled to an Ultra High Pressure Liquid Chromatographer (UPLC) system (Waters nanoAcquity, Manchester, U.K.). Initially, 5 μL of sample were loaded, desalted and concentrated in a BEH C18 trapping column (Waters, Manchester, U.K.) with the instrument operated in positive ion mode. The peptides were then separated on a BEH C18 nanocolumn (1.7 μm, 75 μm × 250 mm, Waters) at a flow rate of 300 nL/min using an ACN/water gradient; 1% ACN for 1 min, followed by 0−62.5% ACN over 21 min, 62.5− 85% ACN for 1.5 min, 85% ACN for 2 min and 1% ACN for 15 min.

MS spectra were collected using data-dependent acquisition in the range m/z 400−2,000 using a precursor ion resolution of 30,000, following which individual precursor ions (top 5) were automatically fragmented using collision induced dissociation (CID) with a relative collision energy of 35%. Dynamic exclusion was enabled with a repeat count of 2, repeat duration of 30 s and exclusion duration of 180 s.

#### Protein identification

The ion spectra readings were converted into peak list text files for database search using Proteome Discoverer Software and analysed with two search algorithms: MaxQuant v1.1.1.3624 Andromeda and MASCOT (http://www.matrixscience.com), against the Actinopterygii SwissProt database 57.15. The use of multiple search engines has been shown to expand the number of identified proteins (union) and validates protein identifications (intersection) [43]. The initial search parameters allowed for a single trypsin missed cleavage, carbamidomethyl modification of cysteine residues, oxidation of methionine, acetylation of N-terminal peptides, a precursor mass tolerance of 10 ppm, a fragment mass tolerance of ±0.5 Da, and a FDR of 0.01. After MASCOT search, only protein hits with score above ≥ 18 were accepted. The Exponentially Modified Protein Abundance Index (emPAI) [44] was also calculated and used for relative quantification among groups.

#### Bioinformatics and gene ontology (GO) analysis

Initially, lists obtained for each group and from the different search engines were combined and redundancies were manually curated. The mean emPAI values, frequency of each protein hit were calculated, and a table containing MW (kDa), highest MASCOT score, matches, highest MaxQuant score was produced (S2 Table). This list was analysed for gene ontology (GO) in STRAP *v 1*.*5*. [42], using UniProtKB and EBI GOA databases, after which secreted (extracellular) proteins were revealed. *Venny 2*.*1*. [45] was used to generate a Venn diagram depicting all detected proteins and secreted (extracellular) proteins between different groups.

## Results

### Peptide analysis on the cephalic secretion of *Arapaima gigas*

**Fig 1:**
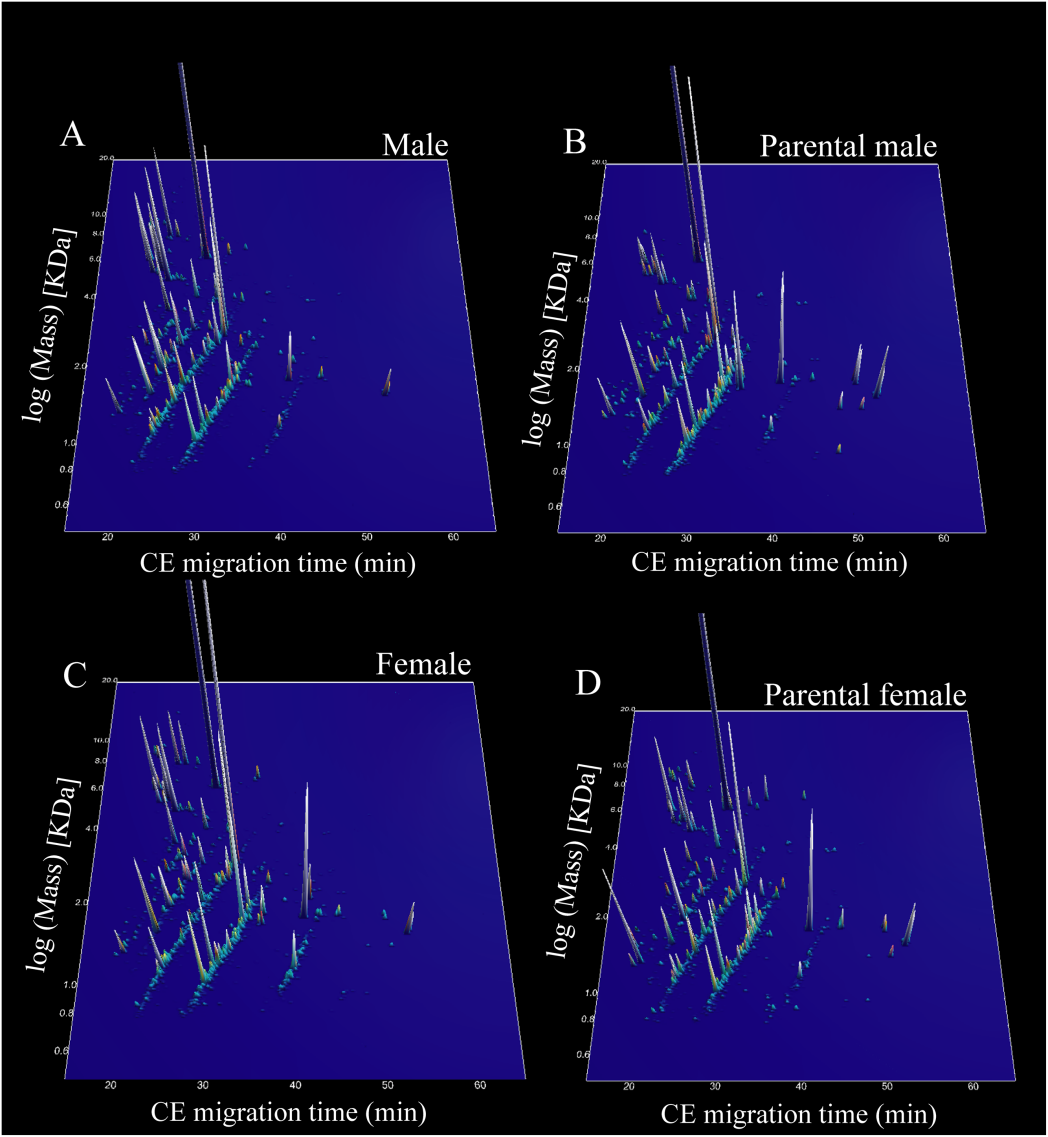
Compiled CE-MS peptide fingerprints from the cephalic secretion of *Arapaima gigas*. A. Non-parental males (males; n = 9). Parental males (PC-males; n = 5). C. Non-parental females (females; n = 11). D. Parental females (PC-females; n = 5). Migration time of capillary electrophoresis (CE) is shown in X axis whereas Y is the logarithmic scale of molecular mass (kDa) and Z is the mean signal intensity.

Based on CE migration time and molecular weight (MW) from individual samples, a compilation of peptide patterns was generated for each studied group (Fig 1). The number of detected peptides (mean ± SD) ranged from 187 to 3005 (1374.1 ± 1017.8; n = 9) in males, from 402 to 1397 (682.8 ± 446.2; n = 5) in PC-males, from 170 to 2243 (755.3 ± 639.9; n = 11) in females and from 202 to 2560 (1008.0 ± 935.2; n = 5) in PC-females. From these peptides, 28 peptides were significantly different between males and PC-males (Wilcoxon P<0.05; BH>0.05) (17 identified). Comparing females and PC-females, 126 peptides were found significantly different (Wilcoxon P<0.05; BH>0.05) (76 identified); 51 peptides were significantly different between males and females (Wilcoxon P<0.05; BH>0.05) (41 identified) and finally, 9 peptides were found significantly different between PC-males and PC-females (8 identified). S1 Table depicts all identified peptides, MW, primary gene name, sequences and group differences.

**Fig 2:**
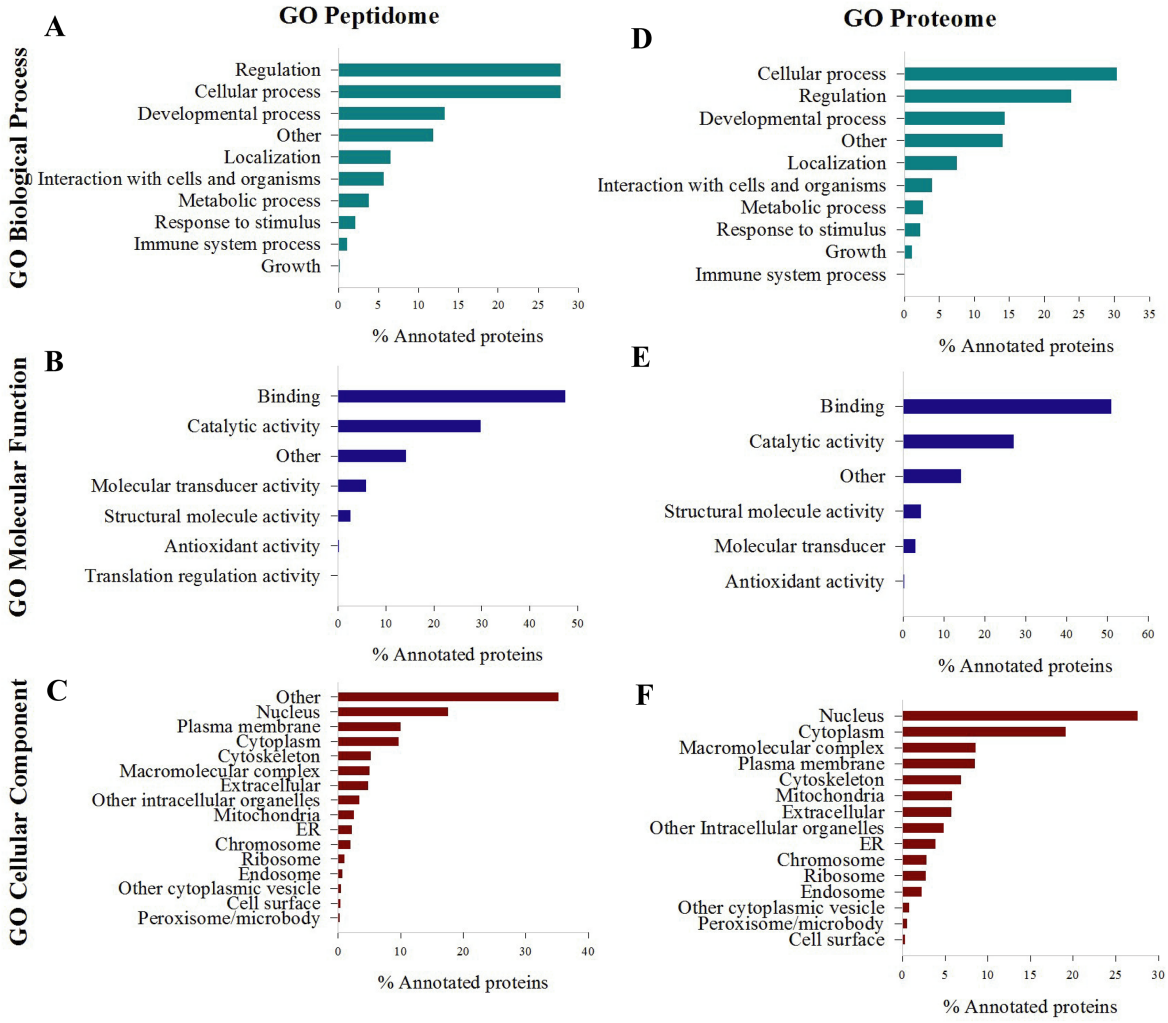
Gene ontology (GO) comparison of peptidome (7009 peptides) and proteome (422 proteins) identified in the cephalic secretion of *Arapaima gigas*. A, B and C. Peptidome GO for biological process, molecular function ad cellular component, respectively. D, E and F. Proteome GO for biological process, molecular function ad cellular component, respectively. Analyses were conducted in STRAP *v*. *1*.*5* [42].

A list with 7009 unique peptide sequences was obtained compiling LC-MS/MS data from all the studied groups. Regarding their GO biological processes, most were related to regulation (1462; 27.8%), cellular process (1461; 27.8%), developmental process (698; 13.3%), localization (345; 6.6%), interaction with cell and organisms (298; 5.7%) and others (625; 11.9%) (Fig 2A). Considering their molecular functions, main GO categories were binding (2364; 47.4%), catalytic activity (1485; 29.8%), molecular transducer activity (292; 5.8%), structural molecule activity (127; 2.5%), antioxidant activity (7; 0.1%) and others (709; 14.2%) (Fig 2B). Considering their GO cellular components, most belonged to the nucleus (767; 17.6%), plasma membrane (438, 10%), cytoplasm (421; 9.6%), cytoskeleton (228, 5.2%), macromolecular complex (219, 5%), extracellular (207, 4.7%), and others (Fig 2C).

### Protein analysis on the cephalic secretion of *Arapaima gigas*

**Fig 3:**
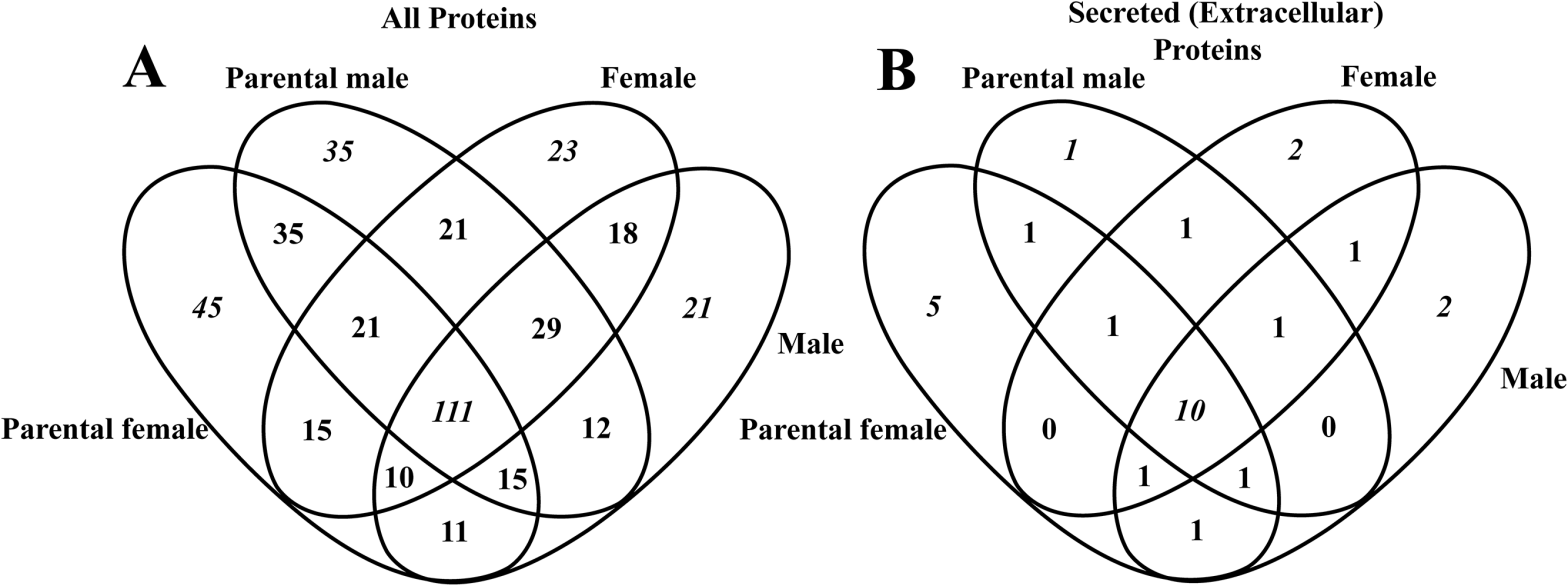
Number of unique and shared proteins in the cephalic fluid of *Arapaima gigas* comparing parental (PC-males) and non-parental males (males), and parental (PC-females) and non-parental females (females). A. Total of 422 proteins catalogued after GeLC-MS/MS and the 28 secreted (extracellular) proteins (B) revealed after GO analysis. Venn diagrams were produced in Venny [45].

The total protein content was not significantly different among males, females, PC-males and PC-females (one-way ANOVA; P>0.05). Total protein ranged from 0.25 to 4.29 μg/μL (1.80 ± 1.35 μg/μL; n = 6) in females, from 0.60 to 16.26 μg/μL (6.23 ± 6.29 μg/μL; n = 5) in PC-females, from 0.39 to 6.88 μg/μL (1.71 ± 2.55 μg/μL; n = 6) in males and from 1.82 to 7.89 μg/μL (4.08 ± 2.59 μg/μL; n = 5) in PC-males. In total, 422 proteins were identified combining all studied groups. A complete list that includes protein names, family, species, molecular weight (MW), scores, matches, mean emPAI values, frequency is catalogued in S2 Table. Aiming to compare the number of proteins unique and shared between groups, a Venn diagram was generated (Fig 3A). PC-males had 35 exclusive proteins whereas PC-females had 45 with both groups sharing other 35 proteins at a parental care condition. Males had 21 exclusive proteins and females 23 with both groups sharing 18 proteins. A total of 111 proteins were common to all groups.

GO analyses were conducted to classify the complete catalogue with 422 proteins, resulting in very similar pattern compared to the peptidome GO analysis (Fig 2). Based on annotations obtained for biological processes, most of the identified proteins are associated with cellular processes (194; 30.5%), regulation (147; 23.1%), developmental process (97; 15.3%), localization (46; 7.2%), interaction with cell and organisms (24; 3.8%) and others (92; 14.5%) (Fig 2D). Considering molecular functions, proteins were mainly associated with binding (245; 51.8%), catalytic activity (116; 24.5%), structural molecule activity (22; 4.7%), molecular transducer (12; 2.5%), antioxidant activity (2; 0.4%) and others (76; 16.1%) (Fig 2E). Considering cellular component GO analysis, most of the proteins belonged to the nucleus (133; 22.9%), cytoplasm (96; 16.5%), macromolecular complex (46, 7.9%), plasma membrane (36, 6.2%), mitochondria (24, 4.1%), extracellular (28, 4.8%) and others (86; 14.8%) (Fig 2F).

### Secreted (extracellular) proteins in the cephalic fluid of *Arapaima gigas*

GO analysis revealed 28 secreted (extracellular) proteins in the different groups (Fig 3B), which are catalogued in Table 1. Their putative functions in the biology of *A*. *gigas* were retrieved from available literature on teleost species and emPAI values used as a measure of relative quantification between studied groups. Two hormones were detected in PF: prolactin (PRL) and stanniocalcin (STC). In all studied groups, proteins related to immunological system processes were identified (Table 1). Among them, proteins with known antibacterial roles and other defence proteins are listed. Serotransferrin (TF) was found up-regulated in PC-males (3.8-fold in relation to males group), α -1-antitrypsin homolog was detected in all groups, apolipoprotein A-I (apoA-I) was present in females, PC-males and PC-females, complement C3 (C3) and complement component C8 beta chain (C8B) were both present in all groups. The remaining secreted proteins are putatively involved in growth (fibroblast growth factor 3 found in PC-females and growth/differentiation factor 6-A in PC-males) brain regulation and development (different Wnt proteins), embryonic development in parental and non-parental fish (chordin, olfactomedin-like protein 3B, laminin subunit gamma-1) and others.

**Table 1:** List of 28 secreted proteins (extracellular) present in the proteome of *Arapaima gigas*. Putative functions, detected groups and relative measure of concentration (emPAI) are given for pools of males (M), females (F), parental care males (PM) and parental care females (PF).

UniProt n°	Name	Hormones	Detected group	emPAI	Reference
O93337	Prolactin	Several roles in parental fish including mucous production and growth	PF	0.16	[28, 46, 47]
Q08264	Stanniocalcin	Prevention of hypercalcemia	PF	0.13	[48, 49]
		**Immune system**			
P84122	Thrombin	Functions in blood homeostasis, inflammation and wound healing	M	1.22	[50]
P79819	Serotransferrin	Role in stimulating cell proliferation. Known to inhibit bacterial colonization in fish	M, F, PM, PF	0.14, 0.15, 0.53, 0.09	[51, 52]
P80426	Serotransferrin-1	Role in stimulating cell proliferation. Known to inhibit bacterial colonization in fish	M, F, PM	0.25, 0.29, 0.18	[51, 52]
P80429	Serotransferrin-2	Role in stimulating cell proliferation. Known to inhibit bacterial colonization in fish	PM, PF	0.21, 0.21	[51, 52]
P32759	α -1-antitrypsin homolog	Identified in carp perimeningeal fluid	M, F, PM, PF	0.14, 0.09, 0.19, 0.09	[53, 54]
Q8JFG3	Tumor necrosis factor	Important mediator in resistance against parasitic, bacterial and viral infections	M, F, PM, PF	0.13, 0.13, 0.13, 0.14	[55]
O42363	Apolipoprotein A-I	Participates in the reverse transport of cholesterol from tissues to the liver	F, PM, PF	0.13, 0.13, 0.13	[56]
Q92079	Serotransferrin	Role in stimulating cell proliferation. Known to inhibit bacterial colonization in fish	M, F, PM, PF	0.11, 0.13, 0.11, 0.07	[51, 52]
P98093	Complement C3	Central role in the activation of the complement system	M, F, PM, PF	0.05, 0.07, 0.07, 0.07	[57]
Q9PVW7	Complement component C8 beta chain	Play a key role in the innate and adaptive immune response	M, F, PM, PF	0.06, 0.06, 0.06, 0.06	[58]
Q3B7P7	Ubiquitin-60S ribosomal protein L40	Ubiquitin A-52 residue ribosomal protein	M, F, PM, PF	–	–
Q9W686	Semaphorin-3ab	Influence pathway choice of extending motor axons along development	M, F, PM, PF	0.04, 0.04, 0.04, 0.04	[59]
		**Growth**			
P48802	Fibroblast growth factor 3	Regulation of cell proliferation, differentiation and embryonic development	PF	0.13	[60]
P85857	Growth/differentiation factor 6-A	Growth factor that controls proliferation and cellular differentiation in the retina	PM	0.08	[61]
		**Brain regulation/development**			
P24257	Protein Wnt-1	Involved in neurogenesis	F	0.09	[62]
P47793	Protein Wnt-4a	Probable brain developmental protein	M, PF, F	0.09, 0.09, 0.09	[63]’
P43446	Protein Wnt-10a	Signalling molecule important in CNS development	M, PF	0.07, 0.07	[64]
Q0P3W2	Olfactomedin-like protein 3B	Secreted scaffold protein with essential role in dorsoventral patterning in early development	PF	0.08	[65]
O57472	Chordin	Developmental protein, dorsalizing factor, somitogenesis	M, F, PM, PF	0.03, 0.03, 0.03, 0.04	[66]
Q1LVF0	Laminin subunit gamma-1	Mediate attachment, migration and organization of cells into tissues in embryonic development	M, PF, PM	0.02, 0.02, 0.03	[67]
		**Others**			
Q6NWB6	Unique cartilage matrix-associated protein	Control of osteogenic differentiation	M	0.24	[68]
B9TQX1	Unique cartilage matrix-associated protein	Control of osteogenic differentiation	PF	0.23	[69]
B0JZP3	Protein THEM6	Thioesterase superfamily member 6	M, F, PM, PF	0.16, 0.16, 0.22, 0.16	[70]
Q7T297	Protein FAM172A		F	0.08	
O93484	Collagen alpha-2(I) chain	Type I collagen is a member of group I collagen (fibrillar forming collagen)	F, M	0.05, 0.03	[71]
Q90X49	Coiled-coil domain-containing protein 80	Promotes cell adhesion and matrix assembly	F, PM	0.04, 0.04	[72]

## Discussion

The lateral line of *A*. *gigas* is an open system like in most teleosts [73, 74]. On the head surface of arapaima, many pore bearing sculptures can be easily recognized, which are the external openings of the interconnected cephalic canals composing the anterior lateral line system. Being an open system, water inflow and fluid outflow occur within these canals (see Coombs, Bleckmann [75] for review). In *A*. *gigas*, this fluid is secreted from the head into the water and its release is intensified during parental care [14], when the secretion remains within range of developing eggs, larvae and offspring. Previous authors have suggested that offspring of *A*. *gigas* would feed from this secreted fluid [14, 18, 76], similarly to fry from several cichlid species feeding their parent’s mucus, which provides nutrients, hormones and passive immunity [4, 77, 78]. In contrast with discus fish (*S*. *aequifasciata*), where the mucus is a source of proteins to the fry [27], in arapaima the relatively low concentration of proteins determined in the cephalic fluid would suggest a minor role as a protein source to offspring. This was also supported by the similar levels of protein found in parental and non-parental care fish. For this reason, this study focused on the differential protein and peptide composition between parental and non-parental care males and females.

Investigation of the parental care condition in *A*. *gigas* is challenging. Collection of samples in the wild is prohibitive since the species is endangered [7] and captive reproduction is still the main bottleneck for aquaculture development hindering further investigations on the possible roles of the cephalic fluid in parental care [8]. In this study, two couples reproduced in the research station allowing the collection of valuable samples from parental fish for the first time in the species. The cephalic fluid of *A*. *gigas* involves a fluid interaction with the endolymph from the inner-ear, which in this study was supported by the presence of the peptide pcdh15b, and with the CSF, supported herein by the presence of peptides related to synapse assembly and neurotransmitter secretion (*i*.*e*. otud4, ribeye a protein, tjp1b and syn1). Also, the canals are composed by the integument, skull bones and cartilage, which has been shown to be highly vascularized and innervated in *A*. *gigas* and also containing integumentary glands potentially secreting peptides and proteins into the cephalic fluid [14, 18], and also the circulatory system, revealed by the previous detection of sex steroids in the cephalic fluid [79]. Given this multiple tissue interactions and the open nature of the cephalic canal system, studies on the cephalic fluid of *A*. *gigas* are also technically difficult. Furthermore, it is also difficult to obtain precise information on fish age, maturity and reproductive condition [80], and these taken together explain the large variability in peptide number and total protein content observed in the cephalic secretions in the present study. Regarding peptide variability, PC-males was the less variable group as denoted by lower SD, reflecting a more consistent physiological condition along parental care.

After CE-MS analysis, significant differences in peptide abundance among the studied groups were detected, and surprisingly, a higher number of peptides were found in PC-females compared to females. This suggests a marked physiological change in PC-females reflected at the peptide level in the secretion, especially given that females are regarded to play a minimal role in parental care after the nest guarding phase [13]. In contrast, when comparing males and PC-males, fewer peptides were identified and most of the identified had reduced abundance in PC-males. As males are known to play a central role in parental care, such findings were surprising. These could indicate the peptide level of the secretion would be of reduced importance along male parental care, though the lack of functional characterisations for the identified peptides also limits such conclusion. In this study, several candidate peptides and proteins were catalogued that will support future comparative studies involving chemical communication and behaviour in parental *A*. *gigas* and other teleosts.

Overall, GO analysis comparing parental and non-parental conditions showed similar results, therefore we report GO analysis combining groups aiming to better characterise the cephalic secretion. When comparing the GO analysis obtained for the proteome and peptidome, also comparable results were seen, with most proteins and peptides being related to biological regulation, cellular and developmental processes. In comparison, very similar results are seen in the murine cochlear sensory epithelia [81], indicating a strict link of the cephalic fluid of *A*. *gigas* with the inner-ear endolymph and/or sensory epithelia comprising the anterior lateral line canals. Comparing the studied groups, parental fish had a higher number of exclusive proteins than non-parental groups, with PC-females again displaying a high number of exclusive proteins suggesting key physiological changes associated with parental care. In total, 422 proteins were identified from the cephalic secretion of parental and non-parental *A*. *gigas*. Given our interest in secreted proteins which could be potentially functional in parental care processes, GO analysis was used and depicted 28 extracellular proteins found present in the cephalic secretion.

The pituitary hormone prolactin (PRL) was detected exclusively in PC-females. PRL is considered a key hormone that has been detected previously in mucus of several teleost females exhibiting parental care behaviour [4, 28]. It also agrees with the increased mucin abundance seen in PC-females. The detection of PRL only in PC-females agrees with a pattern observed in teleosts displaying parental care. In the Midas cichlid (*Cichlasoma citrinellum*), PRL can be actively transferred into the youngs through the mucus feeding, increasing fry growth [4]. In the Amazon discus fish (*Symphysodon aequifasciata*), PRL is also present and upregulated in the mucus of parental females [28]. Other studies with teleosts showed the association of PRL with female reproductive behaviour such as nest building, mouth brooding and the parental care [46]. Given PRL is produced by the pituitary, the likely routes into the cephalic secretion is through the CSF or alternatively, PRL, as mucous component, could be derived from the circulatory system as suggested in other teleosts.

*Stanniocalcin* (STC) is a potent hypocalcemic or antihypercalcemic from bony fishes which in this study was detected in PC-females. STC is synthesized and secreted from the kidney corpuscles of Stannius [49], so its route into the cephalic fluid is very likely through the circulatory system. After spawning in *A*. *gigas*, females undergo a period of rapid ovarian growth leading to repeat spawning during a same reproductive period, since the inter-spawning period appears to be approximately one month (*pers*. *observation*). Presence of STC in PC-females may reflect its proposed role in processes involved in gonadal development [82]. In other fish species, calcium levels (both plasma ionic and total calcium) have been shown to increase in response to oestradiol-17beta (E2), which triggers vitellogenesis [83], while in other studies only increases in total plasma levels of calcium (solely due to binding to vitellogenin) have been shown [84]. In any case, during gonadal development and vitellogenesis calcium influxes are increased [83], and consequently the demand on calcium control mechanisms is higher. Within this context and as suggested by other authors [82] STC may act as hypocalcemic factor acting as a regulator of calcium levels. Whether these changes would impact larvae and fry development remains to be investigated.

Being an open system, the cephalic canals of *A*. *gigas* are a vulnerable door for pathogens and parasites, which could significantly impact adult health. Parasitism and infections in the inner ear and lateral line are indeed serious disease for many teleosts [85]. Therefore, the presence of immune competent proteins to defence against bacteria, viruses and parasites would be expected in the cephalic secretion, and their presence along parental care could be enhanced. Out of the 28 secreted proteins detected, 12 have immunological functions described in teleosts, some of which has been related to parental care. From these, four serotransferrin (TF) species were identified with two present in all studied groups and one with higher abundance (emPAI-based) in PC-males. TF is known to supress iron and therefore inhibits bacterial colonization, playing also an important role in macrophage activation [86]. The importance of TF in immunological defence has been reported in the mucus of gilthead seabream (*Sparus aurata*) [22, 87], sea bass (*Dicentrarchus labrax*) [88], and also within the internal pouch where male seahorse (*Hippocampus* spp.) incubate their fry during early pregnancy [78]. Therefore, presence of TF in the cephalic secretion indicates protective functions similar to mucus role in teleosts, and the observed increase observed in PC-males suggests potential roles into developing offspring of *A*. *gigas*, with mechanisms already studied in other parental teleosts.

Another immune active protein detected in the cephalic fluid of all studied groups was the glycoprotein α-1-antitrypsin homolog (A1AT). A1AT belongs to the serpin family of glycoproteins, involved in the control of blood coagulation, fibrinolysis, complement activation, and inflammation processes [53]. This protein has a marked protective function in carp (*Cyprinus carpio*) perimeningeal fluid [53], and for being upregulated in the mouth mucus where eggs are incubated in parental tilapia females (*Oreochromis* spp.) [54]. A similar protective role for developing fry and offspring of *A*. *gigas* could be at work, although further studies are required to test this hypothesis. Similarly, apolipoprotein A-I (apoA-I) was also detected in the cephalic fluid of PC-males and PC-females. ApoA-I has antibacterial properties previously demonstrated *in vitro* in the striped bass (*Morone saxatilis*) [89] and its activity against pathogens suggested in the mucus of gilthead seabream [87, 90], sea bass [88] and cod (*Gadus morhua*) [29]. Two other proteins involved in complement activation were also detected in all groups: complement C3 (C3) and complement component C8 (C8B). Complement activation system results in the formation of the membrane attack complex (MAC), which kills bacteria by disrupting their membranes [58]. C3 has a central role in phagocytic and immunoregulatory processes [57]. Presence of C3 in the mucus of sea bass has been shown [88] and C3 is upregulated in seabream after probiotic intake [90]. The importance of such a varied number of immune proteins in defence response in adults *A*. *gigas* seems consistent as these were detected in all studied groups.

This study detected two secreted proteins in parental fish related to growth regulation: fibroblast growth factor 3 (FGF-3) in PC-females and growth/differentiation factor 6-A (GDF6) in PC-males. FGF-3 is a protein required for inner-ear induction, patterning and maintenance as demonstrated in early larval stages [60], whilst GDF6 plays a role in later eye development in *D*. *rerio* [91]. Therefore, presence of these proteins in PC-males and PC-females likely reflect inner-ear and eye metabolisms, and absence in males and females groups suggests metabolism changes during parental care which could have association with offspring development in *A*. *gigas*. However, several of these detected proteins have known functions in developing embryos. The presence of these proteins in parental care groups remains therefore to be understood. Future studies of the CSF in teleosts should provide a better understanding for their origin and possible roles within the cephalic secretion. This is the case also for several proteins previously reported to be expressed exclusively in developing embryos. These include reported roles in somitogenesis (*i*.*e*. chordin), mesoderm segmentation (*i*.*e*. wnt inhibitory factor 1) and embryonic brain development (Protein Wnt-10b). If these proteins cannot be produced and secreted from the brain of adult parents, as literature suggests, an alternative parsimonious explanation would involve the embryonic production followed by a later inflow into the open cephalic canals of the adults during parental care. This is plausible since during nest guarding, parents remain with their head constantly inside the nest for egg mass fanning and guarding [13] during which inflow of surrounding embryo proteins could be absorbed by the open cephalic canals, although further behavioural studies are needed. Alternatively, the existence of mouthbrooding in *A*. *gigas* could explain the presence of embryonic proteins in the cephalic fluids. Mouth incubation and transport of eggs and larvae has been reported in *A*. *gigas* [10], although not systematically. Therefore, if not mouthbrooding, this study suggests a very close interaction among parent’s head with brood along parental care, and this is in strong agreement with behavioural observations available for the species.

Since the collection of cephalic fluid samples is a relatively non-invasive method, and the fluid has biochemical components from the lateral line system, inner ear endolymph, blood plasma, CSF and skin mucus, application of CE-MS would potentially be a suitable method for health diagnosis as already applied in humans and other mammals if sample collection could be standardised [92, 93]. Other applications could include the search for gender and sexual maturity biomarkers which are considered as critical problems for the captive reproduction of *A*. *gigas* [8], or markers for behavioural investigations.

## Conclusions

For the first-time an investigation shows application of proteome and peptidome techniques to survey cephalic fluids from the anterior lateral line of a teleost species. Analyses in *A*. *gigas* showed sample variability, and a proteomic composition influenced by components from the cephalic canals, inner-ear endolymph, CSF and circulatory system. This study enhances information on the biochemistry of the lateral line system, opening research possibilities in fish physiology and chemical communication. Data from this study highlight the complex role that the cephalic secretion of *A*. *gigas* may play not only on the adults but also on the development of the fingerlings. Previous work suggested fingerlings raised under parental care condition would have higher survival rates and enhanced growth performance compared to in-door reared ones [19]. Although the present study does not confirm such observations it does indicate the importance of parental care strategies not only on survival *per se* but also on fingerling condition which could be positively improved by being in contact with the cephalic secretion.

## Supporting information

S1 Table List of identified peptides through CE-MS. (XLSX)

S2 Table List of identified proteins through GeLC-MS/MS. (XLSX)
